# TRAF2 promotes M2-polarized tumor-associated macrophage infiltration, angiogenesis and cancer progression by inhibiting autophagy in clear cell renal cell carcinoma

**DOI:** 10.1186/s13046-023-02742-w

**Published:** 2023-07-06

**Authors:** Yawei Xu, Lei Li, Wuping Yang, Kenan Zhang, Zedan Zhang, Chaojian Yu, Jianhui Qiu, Lin Cai, Yanqing Gong, Zheng Zhang, Jingcheng Zhou, Kan Gong

**Affiliations:** Department of Urology, Peking University First Hospital; Institute of Urology, Peking University; Beijing Key Laboratory of Urogenital Diseases (Male) Molecular Diagnosis and Treatment Center, National Urological Cancer Center, Beijing, 100034 China

**Keywords:** ccRCC, M2 macrophage, TRAF2, Autophagy

## Abstract

**Background:**

The management of advanced clear cell renal cell carcinoma (ccRCC) remains a major challenge in clinical practice, and the construction of more reliable prognostic prediction models and the further elucidation of key molecular mechanisms of tumor progression are topics in urgent need of in-depth investigation.

**Methods:**

We used CIBERSORT to estimate the proportion of 22 tumor-infiltrating immune cell types in the TCGA-KIRC cohort. Weighted gene co-expression network analysis, least absolute shrinkage and selection operator regression analysis were used to build risk prediction models. Expression patterns and clinical significance of TRAF2 were determined through bioinformatics analysis, real-time qPCR, Western Blot, immunohistochemistry. GSEA analysis, transmission electron microscopy, 2D/3D colony formation assay, cell migration and invasion assay, and tube-formation assay were used to investigate the underlying function and mechanism of the TRAF2/M2 macrophage/autophagy axis.

**Results:**

We constructed a novel prognostic prediction model based on M2 macrophage-related genes, which was identified as an accurate, independent and specific prognostic risk model for ccRCC patients. A reliable nomogram was constructed to predict 1-, 3-, and 5-year overall survival for patients with ccRCC. As one of the constituent genes of the risk model, TRAF2 was determined to be upregulated in ccRCC and associated with poor clinical prognosis. We found that TRAF2 promotes malignant progression of ccRCC by regulating macrophage polarization, migration and angiogenesis. Mechanistically, we found that TRAF2 promotes the polarization of M2 macrophages, and this chemotaxis is achieved in an autophagy-dependent pathway. Orthotopic tumor growth assay results revealed that TRAF2 plays a key role as a promotor of ccRCC growth and metastasis.

**Conclusions:**

In conclusion, this risk model is highly predictive of prognostic in ccRCC patients, which is expected to promote improved treatment evaluation and comprehensive management of ccRCC. Moreover, our findings reveal that the TRAF2/M2 macrophage/autophagy axis plays a key regulatory role in the malignant progression of ccRCC, and suggest that TRAF2 is a potential novel therapeutic target for advanced ccRCC.

**Supplementary Information:**

The online version contains supplementary material available at 10.1186/s13046-023-02742-w.

## Introduction

Renal cell carcinoma (RCC) is one of the ten most common malignancies in both men and women worldwide, accounting for 4.2% of all new cancer cases. The most common histologic subtype of clear cell RCC (ccRCC) arises from the proximal renal epithelial tubules and is responsible for the majority of cancer-related deaths [1, 2]. It is difficult to detect early, resulting in about 30% of ccRCC patients having developed distant metastasis at the time of diagnosis. In recent years, the clinical application of targeted therapy and immunotherapy has improved the overall survival of patients to a certain extent [3]. However, the vast majority of patients will inevitably progress to the stage of metastatic ccRCC due to inter-individual differences in treatment response and drug resistance, and the 5-year survival rate of patients is less than 10% [4]. Therefore, it is crucial to elucidate the molecular drivers of ccRCC progression and metastasis and to develop effective treatment for advanced RCC.

The tumor microenvironment (TME) on which malignant solid tumors depend mainly includes tumor-associated immune cells, tumor-associated fibroblasts, endothelial cells, and extracellular components (growth factors, hormones, extracellular matrix, cytokines, etc.) [5]. TME plays a key role in the maintenance of tumor growth, invasiveness, malignant progression and responsiveness to drugs [6]. Tumor-associated macrophages (TAMs), recruited from circulating monocytes and infiltrating tumor tissue, are the most abundant tumor-infiltrating immune cell population in the TME [7]. Macrophages are highly plastic, and there are two main phenotypes: the classically activated M1 subtype and the alternately activated M2 subtype. M1-type macrophages can exert anti-tumor effects by producing active factors and pro-inflammatory cytokines such as interleukin 6 (IL-6), IL-12, and interferon-γ (IFN-γ) active. Conversely, M2-polarized macrophages exert tumor-promoting activity by producing anti-inflammatory and immunosuppressive cytokines such as IL-13 and transforming growth factor-β (TGF-β) [8]. M2-polarized TAMs promote tumor growth, angiogenesis, and metastasis, and was associated with malignant progression and poor progression in many human tumors [9–11]. Patients with high M2-polarized TAMs infiltration had poorer overall and progression-free survival compared to those with low M2-polarized TAMs infiltration [12], indicating that M2-polarized TAMs are potential biomarkers and promising targets for immunotherapy in ccRCC.

Tumor necrosis factor (TNF) receptor-associated factor-2 (TRAF2) is one of the members of the TRAF superfamily protein, which is an intracellular junction protein with E3 ligase activity. TRAF2 mediates and regulates the activation of nuclear factor kappa B (NF-κB) and microtubule-associated protein kinase (MAPK) signaling pathways by binding to TNFR family proteins. Recent studies have demonstrated that TRAF2 regulates tumor progression by through multiple pathways [13–16]. In lung cancer, TRAF2 mediated a positive feedback loop between macrophages and cancer cells that drives tumor growth [13]. In breast cancer, overexpression of TRAF2 enhanced malignant migration of tumor cells and osteoclast formation, thereby promoting breast cancer osteolytic metastasis [14]. In liver cancer, low expression of TRAF2 was associated with an unfavorable prognosis, and loss of TRAF2 can induce caspase-8 hyperactivation and impaired NF-κB activation, promoting the tumorigenesis and progression of hepatocellular carcinoma [15]. However, the expression pattern of TRAF2 in ccRCC and its impact on patient prognosis and tumor progression are unknown.

In this study, the differential expression of M2 macrophage-related genes (M2 MRGs) was screened out by microarray expression data, and then co-expression analysis, correlation analysis, and enrichment analysis were performed to obtain macrophage-related co-expression networks and enrichment pathways. We then constructed a novel prognostic risk stratification model for ccRCC. We found that the expression of TRAF2 was significantly different in different risk stratifications. Our results showed that TRAF2 was abnormally overexpressed in ccRCC and was associated with a worse patient prognosis. More importantly, our study reveals that TRAF2 promotes the interaction between macrophages and tumor cells in an autophagy-dependent pathway, thereby promoting the malignant progression of ccRCC.

## Materials and methods

### The landscape of immune cell infiltration

CIBERSORT is a deconvolution algorithm based on the principle of linear support vector regression, which uses the expression values of 547 marker genes to characterize the composition of immune cells in tissue samples[17]. We used CIBERSORT to estimate the proportion of 22 tumor-infiltrating immune cell types in The Cancer Genome Atlas (TCGA)-KIRC cohort. All data were normalized and permutation was set to 1000 for stable results [18].

### Co-expression network constructions

We used the weighted gene co-expression network analysis (WGCNA) package to analyze the TCGA-KIRC expression data based on the expression level of M2 macrophages in the samples, and obtained the cluster of genes most correlated with the abundance of M2 macrophages [19]. Briefly, we first converted the expression level of each transcript into a matrix of similarities, and converted the matrix into a topological overlap matrix according to the optimal soft thresholding parameter. Then, assign similar expression patterns to corresponding modules, and find the module with the highest correlation with M2 macrophage content after module and similarity analysis.

### Construction of predicting model and nomogram

The ccRCC samples of the TCGA-KIRC cohort were randomly divided into training and validation sets. Least absolute shrinkage and selection operator (LASSO) regression analysis was performed using the R package “glmnet”. This analysis was used to build a risk prediction model because of its variable screening and complexity adjustment while fitting a generalized linear model. The riskscore = gene A expression level* Coef A + gene B expression level* Coef B + … + gene N expression level* Coef N [20]. The Kaplan-Meier method was used for survival analysis, and the log-rank test was used for comparison of survival between groups. Receiver operating characteristic (ROC) was analyzed by R package “survivalROC” and calculated the corresponding area under the curve (AUC). Based on the risk score and clinical factors (age, sex, grade, stage), the R package ‘rms’ was used to construct a novel nomogram for prognostic prediction. The nomogram can predict the overall survival (OS) of ccRCC patients at 1, 3, and 5 years after diagnosis. Calibration curve was used to assess the agreement between actual data and model predictions.

### Specimens and cell culture

All patient tissue samples were obtained from the Department of Urology, Peking University First Hospital after signing informed consent. Pathological results were diagnosed by at least two senior pathologists from Peking University Institute of Urology. The HK2, OSRC2, 786O, 769P, CAKI2, A498, ACHN, CAKI1, THP1 and HUVECs cells were purchased from the American Type Cultrue Collection (ATCC). All cells were cultured in DMEM (Gibico 11965118) or Endothelial Cell Medium (ScienCell 34,469) with 10% fetal bovine serum (VivaCell 2204031) and 1% penicillin-streptomycin (Procell PB180120). All cells were cultured in an incubator at 37℃ with 5% CO_2_.

### RNA extraction and RT-qPCR

Total RNA was extracted from cells using TRIzol reagent (Invitrogen) according to the manufacturer’s instructions. Reverse transcription of total RNA was performed using a M-MLV reverse transcription kit (Invitrogen). RT-qPCR analysis was performed using SYBR Green PCR Master Mix (Roche) with the Agilent Technologies AriaMx Real-Time PCR System (Agilent). GAPDH served as a normalized internal control and the relative expression was calculated using the 2 ^(−delta delta threshold cycle)^ (2^−∆∆Ct^) method.

### Western blot analysis and antibodies

Western blot analysis was performed according to our previously described protocol [21]. Primary antibodies included anti-TRAF2 (1:1000, Cell Signaling Technology 4724), anti-VEGFA (1:1000, Santa Cruz Biotechnology sc-7269), and anti-β-actin (1:2000, Proteintech 81115-1-RR).

### Immunohistochemistry (IHC) staining

Tumor or paracancerous tissue specimens were fixed overnight in 4% paraformaldehyde and paraffin embedded followed by cut to a thickness of 4 μm. Lung tissues from xenograft mice were stained with hematoxylin and eosin (H&E) to determine the number of metastases. The IHC score was determined by multiplying the staining intensity score (negative = 0, weak = 1, moderate = 2, strong = 3) with the positive rate score (negative = 0, (1–25%) = 1, (26–50%) = 2, (51–75%) = 3, (76–100%) = 4) as described in previous studies [22]. IHC scores of tumor sections ≥ 6 points were defined as “high expression” group.

### Plasmids and transfection

Plasmids and lentiviruses of TRAF2-depleted short hairpin RNAs were provided by OBiO Technology (Shanghai) Corp., Ltd. All constructed plasmids were transfected into cells using Lipofectamine 3000 (Invitrogen) according to the manufacturer’s instructions.

### Transmission electron microscopy (TEM)

TEM assay was performed as described in our previous study [21]. In brief, the cells were sequentially fixed in 1.5% glutaraldehyde, 1% osmium tetroxide, then followed by dehydration of the sample in increasing concentrations of ethanol (50%, 70%, 90%, and 100%) and anhydrous acetone. The samples were dried to complete the polymerization process and cut into 60 nm ultrathin sections (LEICA EM UC7). Sections were stained with 1% uranyl acetate and imaged using a JEM-1400Flash (JEOL, Japan) transmission electron microscope.

### 2D colony formation assay

OSRC2 and CAKI2 cells were seeded in 6-well plates at a density of 500 per well, and then cultured for 10–14 days. After the colony grew to a sufficient size, it was fixed in 4% paraformaldehyde for 20 min, and then stained with 0.1% crystal violet (Beyotime, China). Stained colonies were imaged and manually counted.

### 3D anchorage-independent growth assay

10,000 cells were planted on DMEM with 0.4% low gelling agarose (Sigma-Aldrich, A9414) onto bottom layer (DMEM with 1% low gelling agarose). The medium on the upper layer was replaced every 2 days, and when the 3D colony grew to a sufficient size, the colonies were stained with iodonitrotetrazolium (Sigma-Aldrich, 146-68-9) solution for photographing.

### Cell migration and invasion assay

1–5 × 10^4^ overnight starved cells were seeded into the upper chamber (Corning 3422) with (invasion assay) or without (migration assay) Basement Membrane Matrigel (Beijing Lablead Biotech Co., Ltd, MG6234). DMEM with or without 10% FBS was added to the lower and upper chambers, respectively. 100 nM of 10,058-F4 (MedChemExpress, USA) was added to inhibit cell proliferation. Cells were fixed with 4% paraformaldehyde after 24 h of culture. The cells were stained with 0.1% crystal violet solution and counted manually.

### Tube-formation assay

Spread the Matrigel into 96-well plates at 50 µl per well and leave for 30 min in a cell incubator at 37℃. 10,000 HUVECs cells were added to the microwells and incubated for 6 h. An inverted microscope was used for imaging, and the number of nodes per branch was counted using ImageJ (National Institutes of Health, USA).

### Orthotopic tumor growth

5 × 10^5^ OSRC2-luciferase cells stably expressing shCtrl or shTRAF2#1 were resuspended in 20 µl DMEM with 2% FBS and injected into the mice kidney capsule. Mice were anesthetized and injected intraperitoneally with D-luciferin, and tumor growth was detected using a small animal imager (IVIS-50 chamber, Caliper Life Sciences, USA).

### Statistical analysis

Statistical analyses were performed using SPSS v23.0 (IBM Corp., Armonk, NY, USA) or GraphPad Prism v7.0 (GraphPad Software, La Jolla, CA, USA). Data are presented as the mean ± standard error of the mean (SEM). Univariate and multivariate Cox regression analyses were performed to examine independent factors, A *p* value of < 0.05 was considered significant.

## Results

### Identification of M2 macrophage-related differently expressed genes and functional annotation

We first analyzed the expression profiles of 22 types of tumor-infiltrating immune cells in the TCGA-KIRC cohort using the CIBERSORT algorithm to assess the abundance of immune cell subsets in all samples. We then screened for samples with p values < 0.05 and calculated the proportion of tumor-infiltrating immune cell subtypes in each sample (Table S1). According to the filter conditions the absolute value of Pearson correlation coefficient greater than 0.4, and *p* value less than 0.001, co-expression analysis identified 60 M2 MRGs (Table S2).

**Fig. 1:**
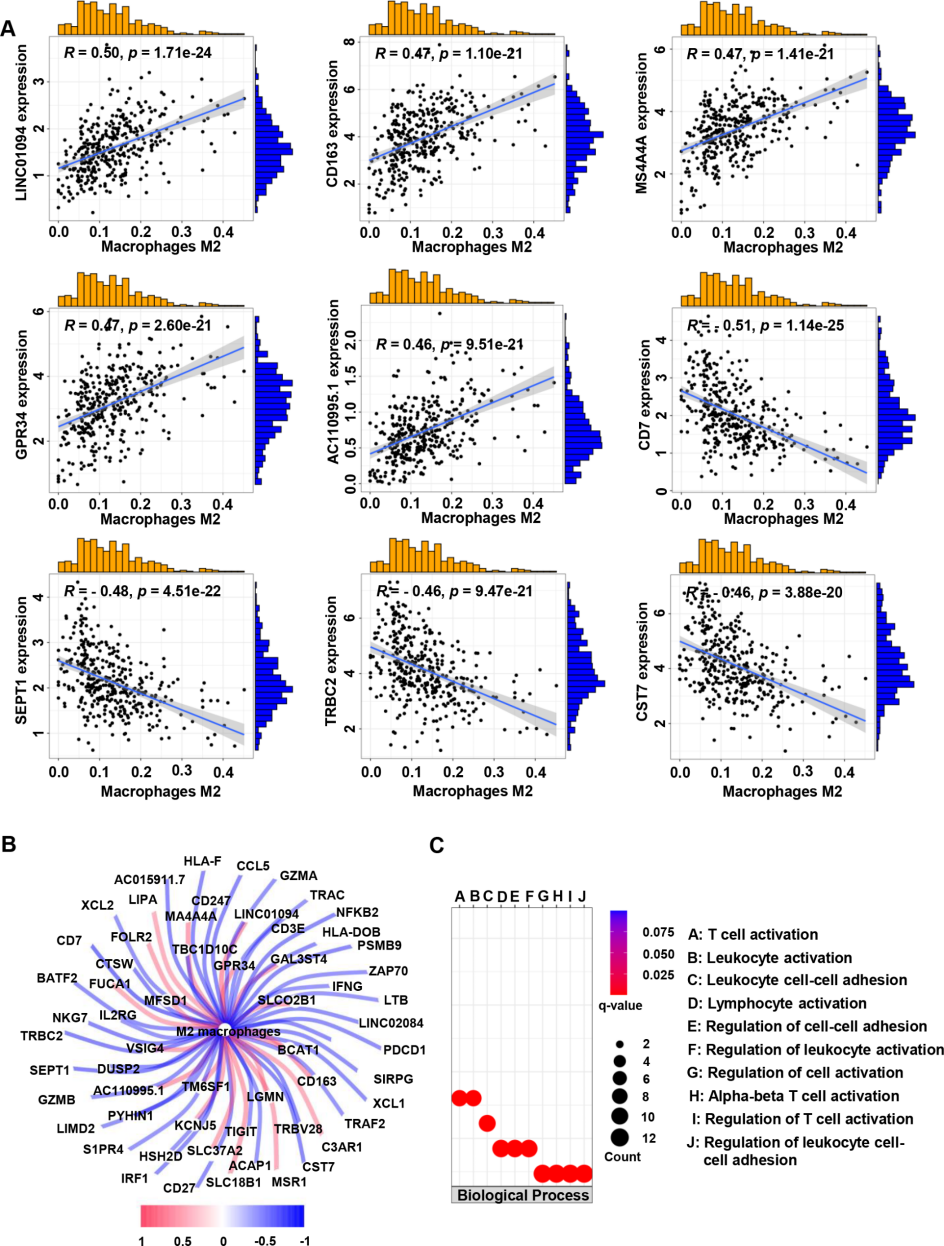
Identification of M2 macrophage-related differently expressed genes (DEGs) and functional annotation. (**A**) Representative of M2 macrophage-related genes (M2 MRGs, top nine ranked by correlation coefficient). (**B**) The regulatory network between M2 MRGs and M2 macrophages. (**C**) GO enrichment analysis for M2 MRGs

The nine genes most correlated with M2 macrophages are shown in Fig. 1A. The regulatory network between M2 MRGs and M2 macrophages was shown in Fig. 1B and Fig. S1. Gene ontology (GO) analysis showed that M2 MRGs were mainly enriched in biological processes related to immune infiltration (Fig. 1C), including T cell activation, leukocyte activation, leukocyte cell-cell adhesion, lymphocyte activation, regulation of cell-cell adhesion, regulation of leukocyte activation, regulation of cell activation, alpha-beta T cell activation, regulation of T cell activation, regulation of leukocyte cell-cell adhesion.

### Construction and validation of a M2 MRGs-based risk prediction model

**Fig. 2:**
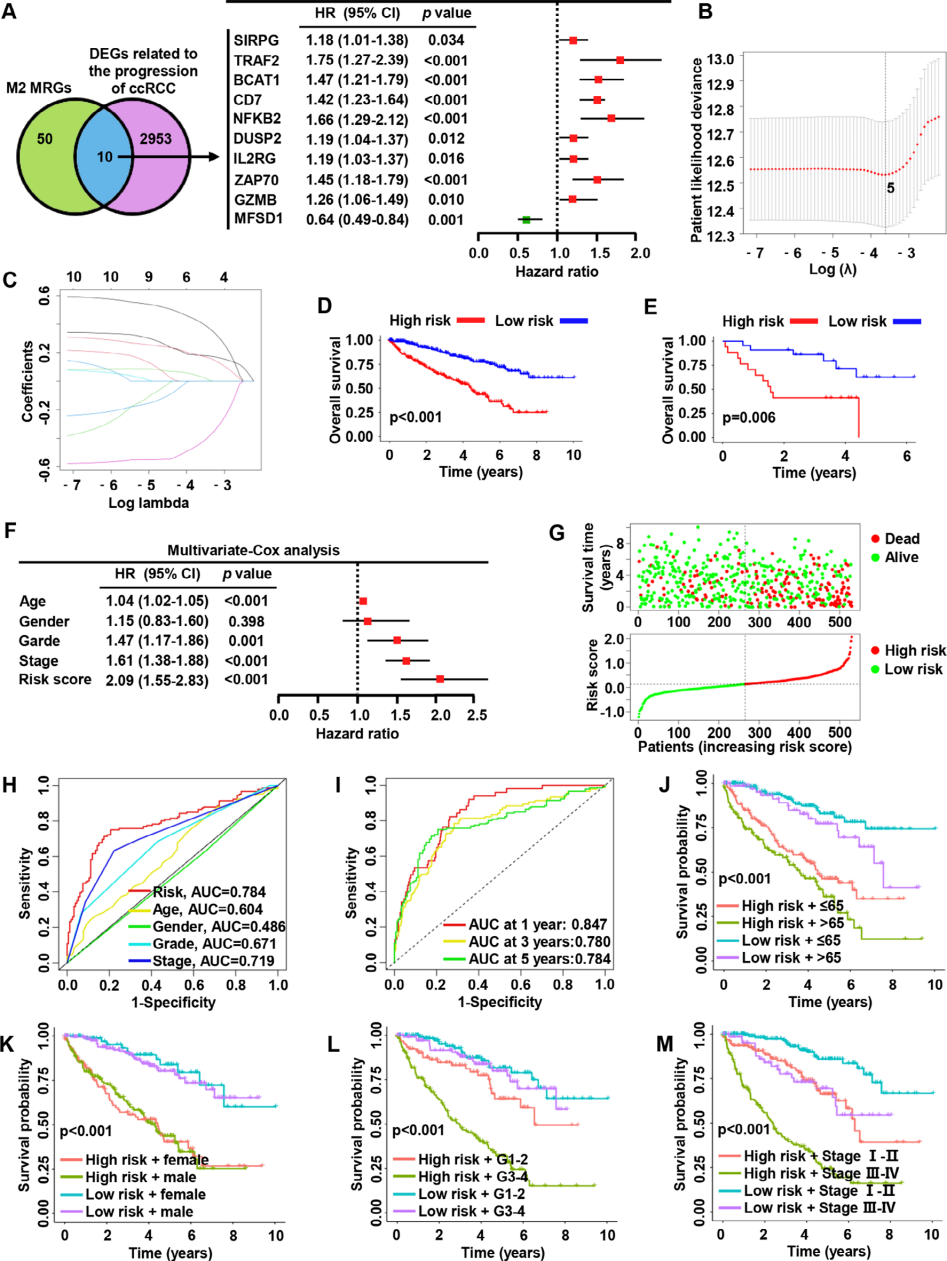
Construction and validation of a M2 MRGs-based risk prediction model. (**A**) A venn diagram between M2 MRGs and differentially expressed genes (DEGs) (left panel), and univariate-Cox regression analysis of cross-set genes (right panel). (**B**) Least absolute shrinkage and selection operator (LASSO) regression analysis determines the number of factors to build a model. (**C**) Partial likelihood bias coefficient distribution plot for selected genes. (**D**) The overall survival (OS) status of the internal validation set. (**E**) The OS status of the external validation set. (**F**) Multivariate-Cox regression analysis of our model’s risk score for ccRCC patients. (**G**) Plots of risk score and survival status distribution. (**H**) Receiver operating curve (ROC) analysis compared the prognostic predictive ability of risk score and clinical factors. (**I**) Time-dependent ROC analysis of 1-, 3-, and 5-year survival for ccRCC patients. (**J**-**M**) Survival analysis of our constructed model combined with clinical factors (age, gender, grade, and stage)

GSE85258 is a dataset exploring differentially expressed genes and molecular mechanisms between primary and metastatic RCC tumors. We reanalyzed data from transcriptome sequencing of 14 patient-paired RCC primary and lung metastases tumor samples. The results of differential analysis showed that the expression levels of 2968 genes were significantly different between the two groups, and we named this group as differentially expressed genes (DEGs) related to ccRCC progression. We intersected ccRCC progression-related genes with M2 MRGs and found 10 genes co-existed in both gene sets (Fig. 2A). Interestingly, univariate-Cox regression analysis showed that all 10 intersection genes were associated with patient prognosis in the TCGA-KIRC cohort (Fig. 2A). Assessing and predicting the prognosis is the key to the management of cancer patients, which is of great significance to the formulation of diagnosis and treatment decisions and the evaluation of efficacy. However, the competence of previously developed markers and predictive models is unsatisfactory, and they are mostly based on clinical and laboratory factors (such as hypercalcemia, performance status) [23, 24]. Therefore, we constructed a patient risk prediction model based on the M2 MRGs for ccRCC patients. The TCGA-KIRC cohort was selected as the training set, and we utilized LASSO regression analysis for dimensionality reduction. Finally, the five M2 MRGs (TRAF2, CD7, MFSD1, NFKB2, BCAT1) were identified as the best candidates for feature genes (Fig. 2B, C). We calculated the risk score with the following formula: (0.0313 × TRAF2 expression) + (0.1818 × CD7 expression) + (−0.4328 × MFSD1 expression) + (0.1790 × NFKB2 expression) + (0.4112 × BCAT1 expression). To test the effectiveness of the constructed risk stratification model, we selected the randomly assigned TCGA-KIRC dataset the GSE29609 as internal and external validation sets, respectively. High-risk patients had worse OS compared with low-risk patients (Fig. 2D-E). Multivariate regression analysis showed that the risk score was an independent prognostic marker for ccRCC patients (HR = 2.09, 95% CI, 1.55–2.83, p < 0.001; Fig. 2F). In addition, the proportion of patients dying with the risk score increases, which indirectly increases the credibility of the risk stratification model (Fig. 2G). ROC analysis showed that the predictive power of the model was significantly higher than that of general clinical factors (Fig. 2H). The time-dependent ROC results showed that the AUC for 1-, 3, and 5-year survival of ccRCC patients predicted by the model were 0.847, 0.780, and 0.784 (Fig. 2I). The risk model was combined with age, gender and grade for survival analysis, which all supported that the risk stratification model was stable and reliable (Fig. 2J-L). When the risk model is combined with stage for survival analysis, we found that the prognosis of patients with low risk and stage I-II is better than that of patients with low risk and stage III-IV, and the prognosis of patients with high risk and stage I-II is better than that of patients with high risk and stage III-IV patients (Fig. 2M). We found that the prognosis of patients with high risk and stage I-II was similar to that of patients with low risk and stage III-IV, but this did not compromise the stability and reliability of the risk model. Collectively, these results suggest that the M2 MRGs-based risk prediction model has the ability to accurately and robustly predict the prognosis of ccRCC patients.

### Construction of the nomogram predicting survival for ccRCC patients

**Fig. 3:**
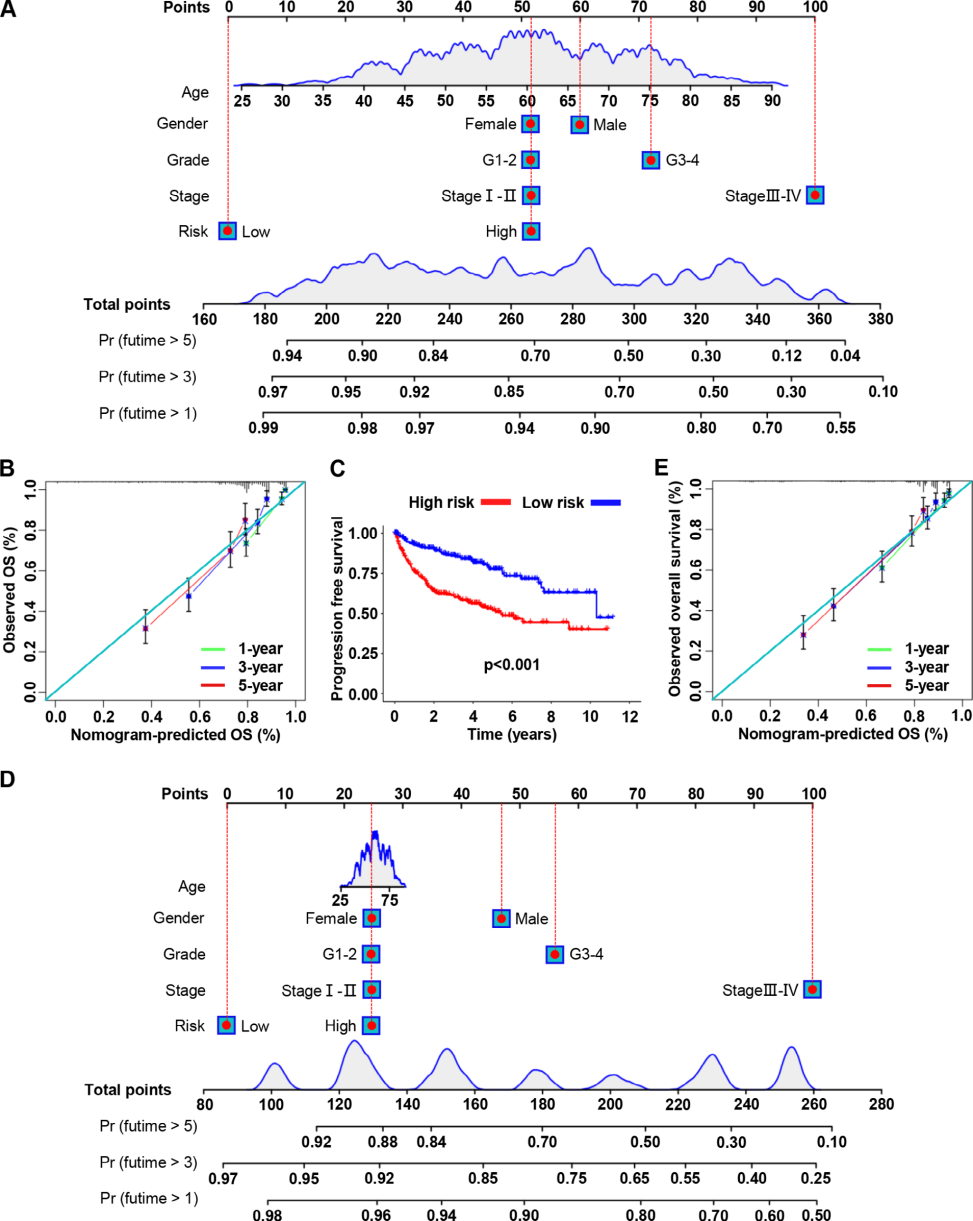
Construction of the nomogram predicting survival for ccRCC patients. (**A**) A novel nomogram containing risk scores for predicting OS in patients with ccRCC. (**B**) The calibration curve shows that the OS predicted by the nomogram fits well with the actual survival. (**C**) The progression-free survival (PFS) status of the internal validation set. (**D**) A novel nomogram containing risk scores for predicting PFS in patients with ccRCC. (**E**) The calibration curve shows that the PFS predicted by the nomogram fits well with the actual survival

The nomogram is a quantitative model for predicting the clinical prognosis of patients. A nomogram was drawn based on the risk score and other clinical characteristics, which allowed the calculation of the OS probability of ccRCC patients at 1, 3, and 5 years (Fig. 3A). Calibration analysis showed that the predicted results were consistent with the actual observations (Fig. 3B). In addition, we found that the progression-free survival (PFS) rate of patients in the high-risk group was lower than that in the low-risk group (Fig. 3C, p < 0.001). A nomogram predicting 1-, 3-, and 5-year PFS of ccRCC patients was constructed (Fig. 3D), and calibration analysis confirmed that the predicted results were consistent with the actual observations (Fig. 3E). Taken together, our results suggest that these prognostic prediction models perform well in predicting the clinical outcomes of ccRCC patients and are expected to be used in the management of clinical patients.

### Upregulated TRAF2 is associated with a poor clinical prognosis in ccRCC

**Fig. 4:**
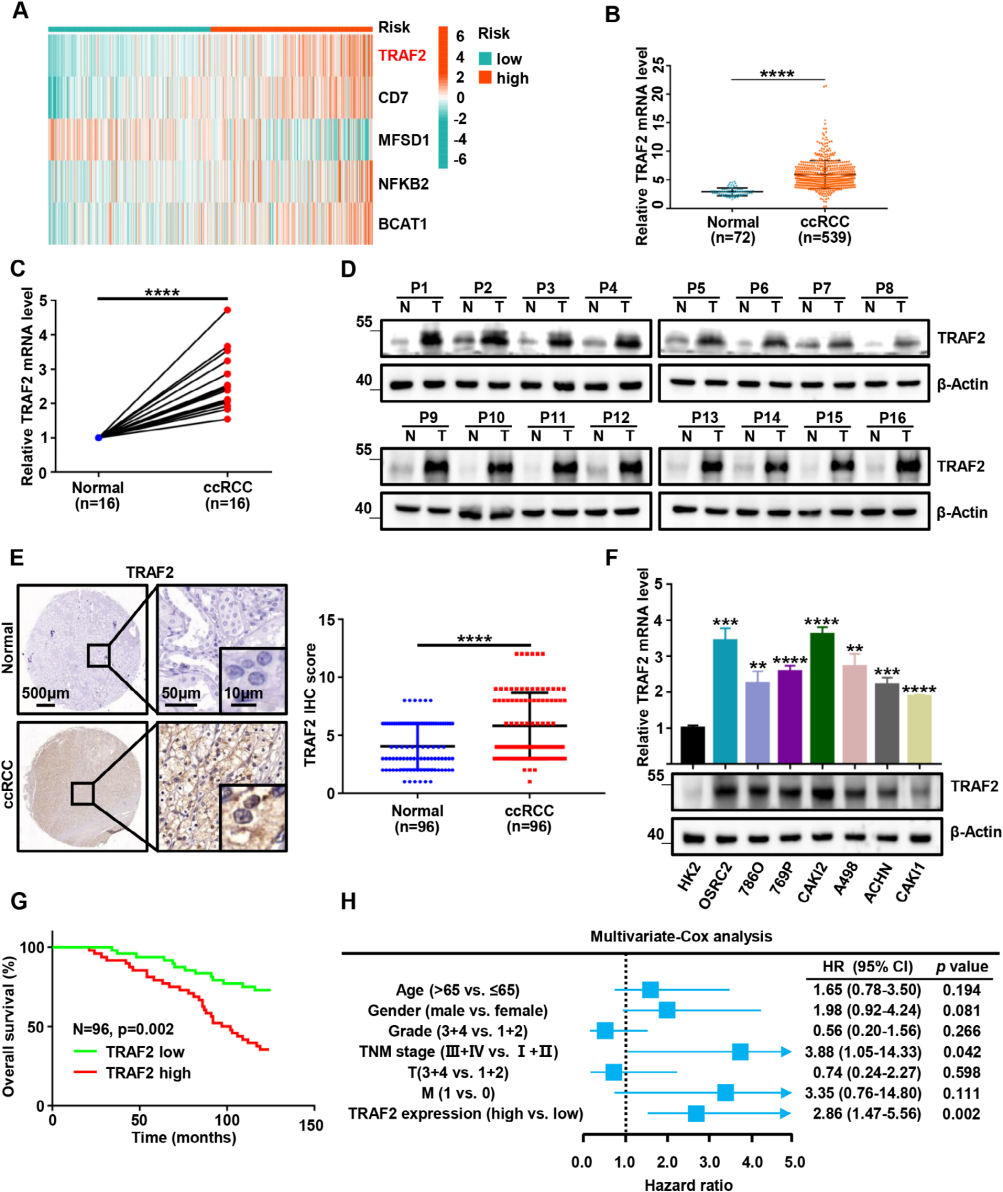
Upregulated TRAF2 is associated with a poor clinical prognosis in ccRCC. (**A**) Heatmap of expression patterns in TCGA-KIRC for the five genes that make up the model. (**B**) *TRAF2* mRNA expression in 539 ccRCC tissues as compared to 72 adjacent noncancerous tissues from TCGA-KIRC data. (**C**, **D**) RT-qPCR and Western Blot analysis of TRAF2 expression in 16 paired ccRCC and adjacent noncancerous tissues. (**E**) Immunohistochemical (IHC) analysis of TRAF2 in a ccRCC tissue microarray (TMA) 96 ccRCC tumor and adjacent noncancerous pairs (left panel) and quantification of TRAF2 expression by IHC analysis in ccRCC TMA samples as compared to normal (right panel). (**F**) Protein and mRNA expression levels of TRAF2 in HK2 and ccRCC cell lines. (**G**) Kaplan-Meier OS analysis of TRAF2 expression in patients with ccRCC (n = 96, p = 0.002, log-rank test). (**H**) Multivariable analyses were performed in the ccRCC cohort. All bars correspond to 95% CIs. Error bars, SEM; **, p < 0.01; ***, p < 0.001, ****, p < 0.0001

First, we analyzed the expression patterns of 5 model constituent genes (TRAF2, CD7, MFSD1, NFKB2 and BCAT1) in the TCGA-KIRC cohort using heatmaps. Interestingly, we found that the differential expression of TRAF2 was most significant in 5 genes and was upregulated in almost all samples of high-risk patients (Fig. 4A). Therefore, TRAF2 was selected to study its role in the progression of ccRCC. Gene expression differential analysis of 539 ccRCC samples and 72 adjacent non-cancer samples revealed that TRAF2 was aberrantly elevated in ccRCC tissues compared with normal tissues (p < 0.0001, Fig. 4B). Subsequently, we validated the total mRNA and protein expression pattern of TRAF2 in 16 paired fresh ccRCC specimens. The results of RT qPCR experiments suggested that TRAF2 was abnormally elevated in ccRCC tissues compared with non-cancerous tissues (p < 0.0001, Fig. 4C). In all sixteen pairs, the protein levels of TRAF2 were found to be higher in human ccRCC tissues than in their adjacent noncancerous tissues by Western Blot (Fig. 4D). We further verified the expression of TRAF2 in a tissue microarray constructed from 96 paired samples. Immunohistochemical staining results showed that TRAF2 was abnormally elevated mainly in the cytoplasm of ccRCC cells (p < 0.0001, Fig. 4E). We then examined the mRNA and protein expression levels of TRAF2 in HK2 (a human renal cortical proximal tubule epithelial cell line) and ccRCC cell lines. Our results showed that the mRNA and protein levels of TRAF2 were higher in ccRCC cell lines than in HK2 cells (Fig. 4F). Kaplan-Meier analysis showed that the patients with high TRAF2 expression had poorer overall 10-year survival than those with low TRAF2 expression (n = 96, p = 0.002, log-rank test, Fig. 4G). Multivariate Cox regression analysis revealed that TRAF2 was an independent predictive marker for the prognosis of patients with ccRCC (HR = 2.86, 95% confidence interval :1.47–5.56, p = 0.002, Fig. 4H). Taken together, these results indicate that TRAF2 is upregulated in ccRCC and TRAF2 might be an independent prognostic factor for patients with ccRCC.

### TRAF2 promotes cell proliferation, migration, and invasion in ccRCC cells

**Fig. 5:**
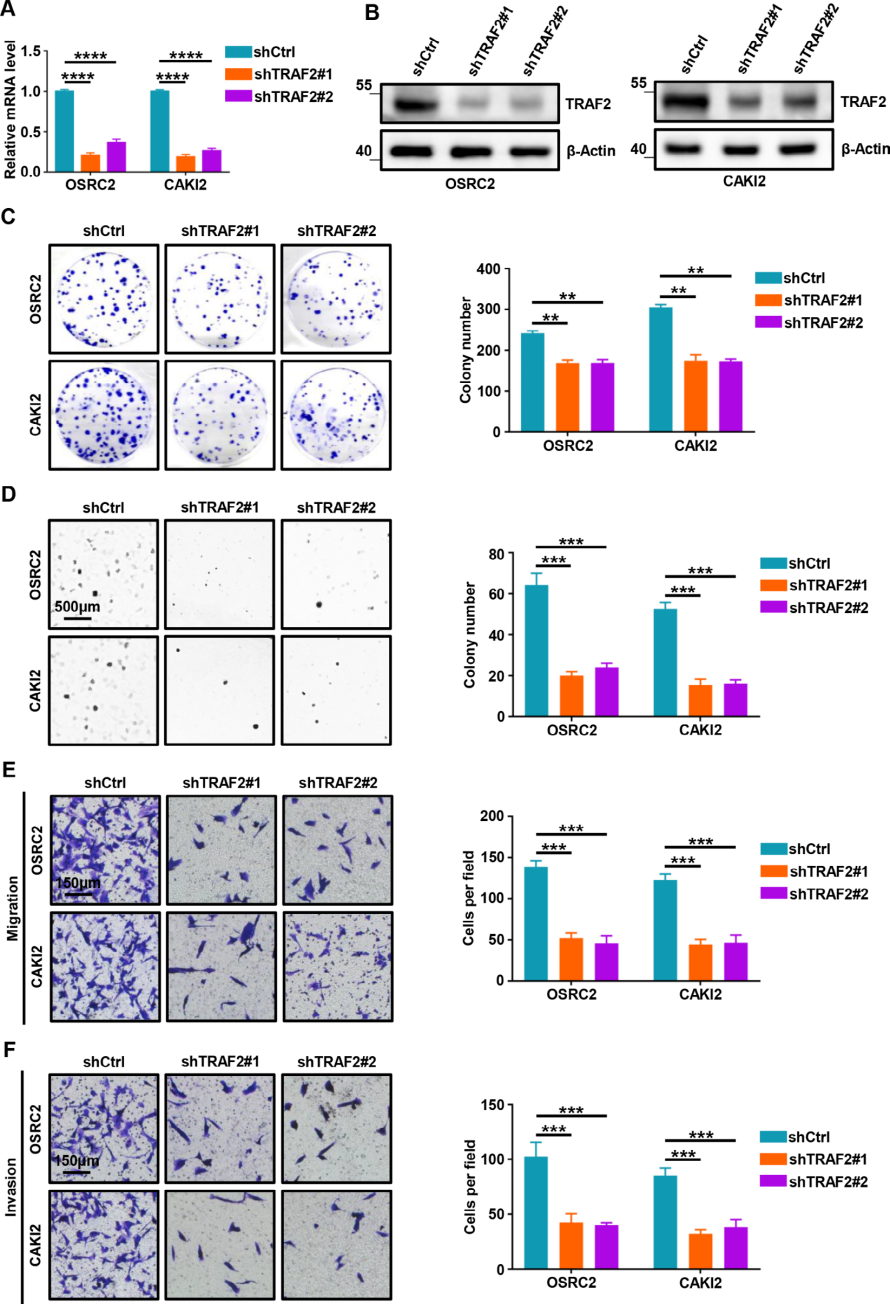
TRAF2 promotes cell proliferation, migration, and invasion in ccRCC cells. (**A**, **B**) Stable knockdown of TRAF2 by shRNAs effectively decreased its both mRNA and protein expression level in OSRC2 and CAKI2 cells. (**C**) Representative images from the colony formation assay (left panel) and colony number analysis (right panel). All experiments were performed in triplicate and data were presented as the mean ± SD. (**D**) Representative 3D soft agar growth pictures (left panel) and quantification of colony number (right panel). (**E**) Representative cell migration pictures (left panel) and quantification of cell migration (right panel). (**F**) Representative cell invasion pictures (left panel) and quantification of cell invasion (right panel). Error bars, SEM; **, p < 0.01; ***, p < 0.001, ****, p < 0.0001

Given that TRAF2 is highly upregulated in ccRCC tissues compared with para-cancerous tissues, we next performed functional experiments to investigate the carcinogenesis of TRAF2 in ccRCC cell lines. We depleted TRAF2 expression in OSRC2 and CAKI2 cells with two independent short hairpin RNAs (shRNAs) (shTRAF2#1 and shTRAF2#2). We confirmed that TRAF2 was stably knocked down in OSRC2 and CAKI2 cells using RT-qPCR (Fig. 5A) and Western Blot assays (Fig. 5B). Firstly, we conducted Cell Counting Kit-8 (CCK-8), two-dimensional (2D) colony growth assays, and three-dimensional (3D) soft agar experiments in two ccRCC cell lines (OSRC2 and CAKI2). Our results showed that TRAF2 depletion by shRNAs led to decreased cell proliferation and colony formation (Fig. S2A-B, Fig. 5C). We also found that TRAF2 depletion led to decreased soft agar growth (Fig. 5D). Next, we conducted migration assays and invasion assays to investigate the effects of TRAF2 depletion on tumor cell migration and invasion potential. Similarly, our results showed that TRAF2 knockdown resulted in decreased migration and invasion capability of tumor cells (Fig. 5E-F). Further, we overexpressed TRAF2 in CAKI1 cells to evaluate the gain-of-function experiments. We confirmed the stable overexpression of TRAF2 in CAKI1 cells using RT-qPCR (Fig. S2C) and Western Blot assays (Fig. S2D). The results of CCK-8 (Fig. S2E), 2D colony, and 3D soft agar growth (Fig. S2F-G) all suggested that TRAF2 promoted ccRCC cell proliferation. More importantly, TRAF2 increased the migration and invasion of ccRCC cells (Fig. S2H-I). Collectively, these results suggest an oncogenic role of TRAF2 in the tumorigenesis of ccRCC.

### TRAF2 promotes ccRCC progression via regulating macrophage polarization, migration, and angiogenesis

**Fig. 6:**
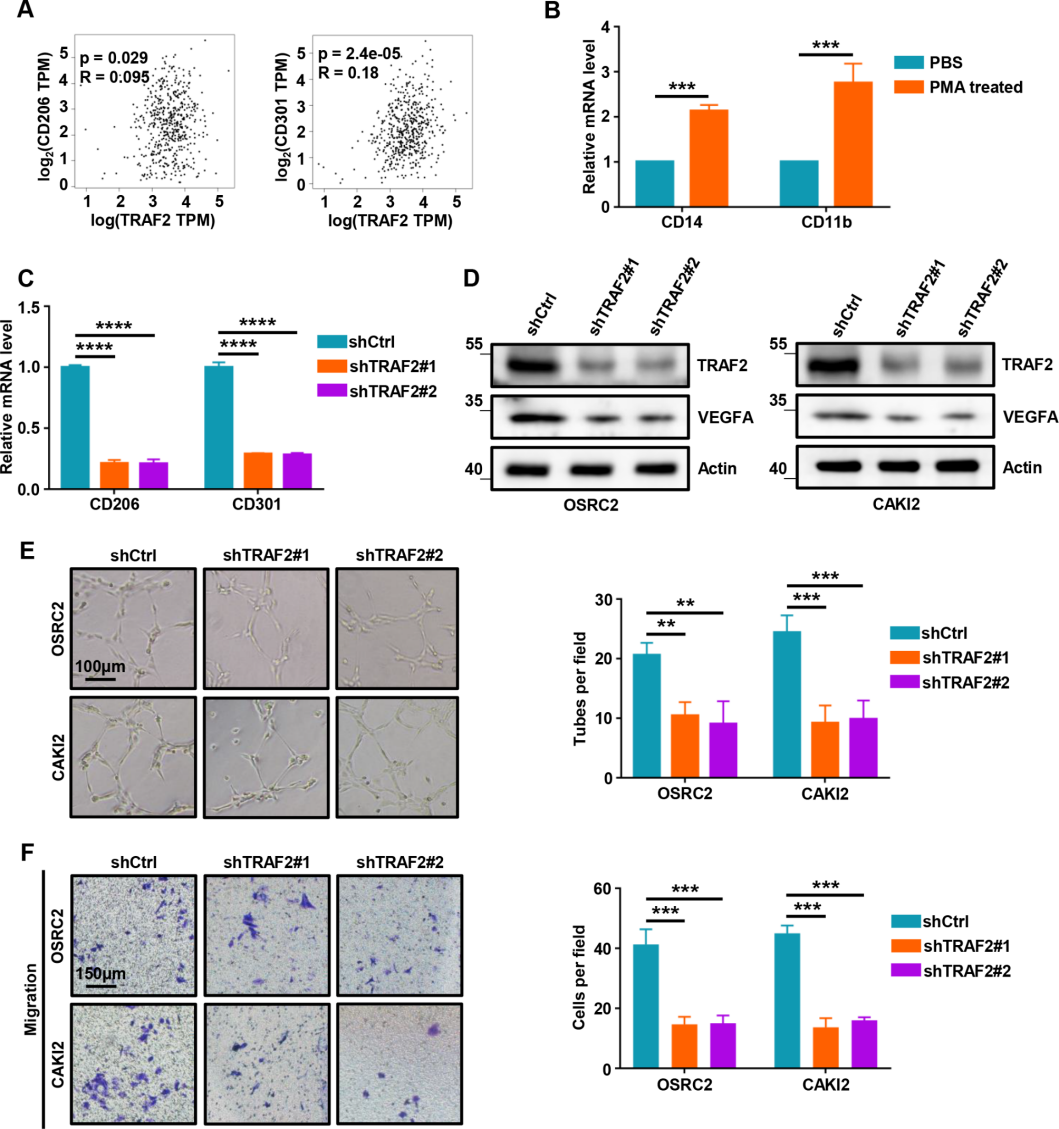
TRAF2 promotes ccRCC progression via regulating macrophage polarization, migration, and angiogenesis. (**A**) Pearson correlation analysis between TRAF2 expression and the markers of M2 macrophages (CD206 and CD301). (**B**) RT-qPCR analysis was used to detect the changes of CD14 and CD11b mRNA expression in THP-1 cells treated with PMA (200 nM, 48 h). (**C**) RT-qPCR analysis was used to detect the markers of M2 macrophages (CD206 and CD301) mRNA expression changes in macrophage-like cells co-cultured with shCtrl or shTRAF2 OSRC2 cells. (**D**) Western Blot analysis was used to detect VEGFA protein expression changes in macrophage-like cells co-cultured with shCtrl or shTRAF2 ccRCC cells. (**E**) Representative tube-formation pictures of in vitro angiogenesis (left panel) and quantification of the node branches (right panel). (**F**) Representative cell migration pictures of macrophages (left panel) and quantification of cell migration (right panel). Error bars, SEM; **, p < 0.01; ***, p < 0.001, ****, p < 0.0001

Since we learned that TRAF2 is a M2 MRG through bioinformatics analysis, we tried to study the possible role of TRAF2 in M2 macrophages promoting tumor progression. As markers of M2 macrophages, M2 macrophages express high levels of CD206 and CD301 compared with M1 macrophages. First, our results showed that the expression of TRAF2 was positively correlated with CD206 (p = 0.029, R = 0.095) and CD301 (p = 2.4e-05, R = 0.18) in 539 TCGA-KIRC tumor samples (Fig. 6A). PMA-stimulated macrophage differentiation of THP-1 monocytes is the most widely used cell model to induce monocyte-macrophage polarization. We used RT-qPCR analysis and flow cytometry (FC) to detect the changes in the expression of macrophage markers CD14 and CD11b in THP-1 cells after 200 nM PMA treatment for 48 h. Our results showed that THP-1 cells were successfully induced to differentiate into macrophage-like cells (Fig. 6B, Fig. S3A-B). To investigate the effects of TRAF2 depletion on macrophage polarization, OSRC2 cells with or without TRAF2 knockdown were co-cultured with macrophage-like cells (Fig. S3C). Interestingly, we found that knockdown of TRAF2 inhibited macrophage polarization towards M2 (Fig. 6C, Fig. S3D-E). The immune microenvironment maintained by macrophages plays an important role in tumor angiogenesis, so we performed Western Blot experiments to detect the effect of TRAF2 depletion on tumor angiogenesis (Fig. 6D). Our results showed that depletion of TRAF2 resulted in decreased VEGFA protein in OSRC2 and CAKI2 cells. HUVECs were cultured in the conditioned medium from co-culture of ccRCC cells with TRAF2 depletion and macrophage-like cells (Fig. S3F). The results of tube formation assay and migration assay showed that TRAF2 depletion resulted in impaired angiogenesis and migration of co-cultured HUVECs cells (Fig. 6E-F). Moreover, TRAF2 overexpression promoted macrophage polarization towards M2 (Fig. S3G-I) and increased the tube formation (Fig. S3J) and migration (Fig. S3K) of HUVECs. Together, these results demonstrate that TRAF2 promotes ccRCC progression via regulating macrophage polarization, migration, and angiogenesis.

### TRAF2 induces macrophage polarization in an autophagy-dependent pathway

**Fig. 7:**
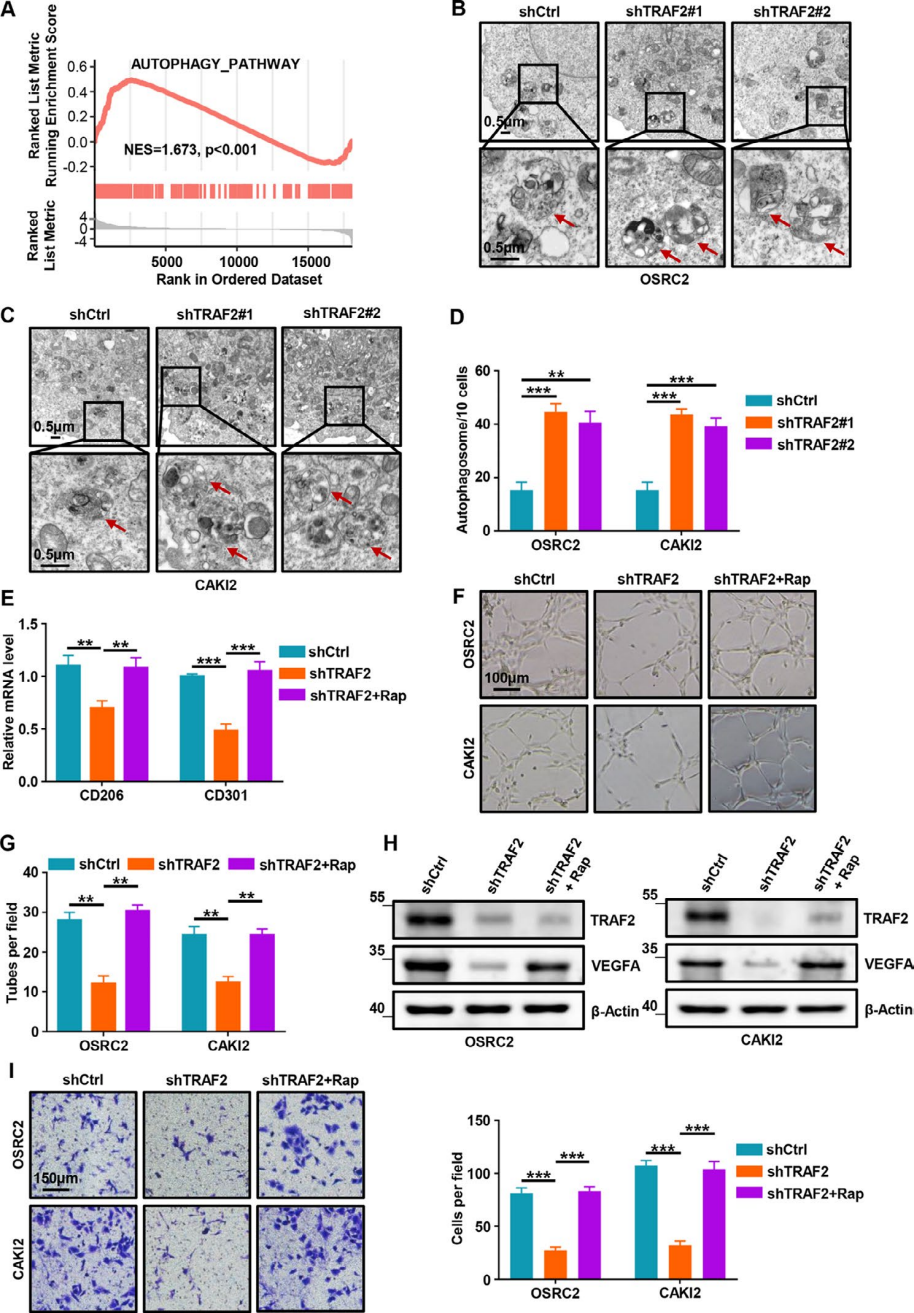
TRAF2 induces macrophage polarization in an autophagy-dependent pathway. (**A**) GSEA analysis was performed in TCGA-KIRC cohort to reveal the association between TRAF2 and the activation of autophagy pathway. (**B**, **C**) Transmission electron microscopy (TEM) demonstrating autophagosomes in shCtrl and TRAF2 depleted OSRC2 and CAKI2 cells. Red arrows indicate autophagosomes. (**D**) Quantification of autophagosomes in cells. (**E**) RT-qPCR analysis was used to detect CD206 and CD301 mRNA expression in macrophage-like cells with rapamycin treatment (100 nM, 48 h). (**F**) Representative tube-formation pictures of in vitro angiogenesis. (**G**) Quantification of the node branches. (**H**) Western Blot analysis of the effect of autophagy activator on the expression of VEGFA protein. (**I**) Representative cell migration pictures of macrophages (left panel) and quantification of cell migration (right panel). Error bars, SEM; **, p < 0.01; ***, p < 0.001

Considering that TRAF2 functions as a ccRCC oncogene and regulates macrophage polarization, further pathway analysis was used to investigate the specific molecular mechanism. GSEA analysis was performed in TCGA-KIRC cohort and results showed that the autophagy signaling pathway was enriched in samples with high TRAF2 expression (NES = 1.673, p < 0.001, Fig. 7A). To investigate the regulation of autophagy by TRAF2, we analyzed the effect of TRAF2 depletion on autophagosome formation in OSRC2 and CAKI2 cells by TEM analysis. Our results showed that TRAF2 depletion led to increased autophagosomes, implying that autophagic activity was activated (Fig. 7B-D). Western Blot assays confirmed that TRAF2 depletion increased autophagy, while TRAF2 overexpression decreased autophagic flux (Fig. S4A-B). Rapamycin is an autophagy activator that can effectively activate the formation of autophagosomes [25]. To determine whether the effect of TRAF2 on macrophage polarization is dependent on autophagy regulation, we treated TRAF2-depleted cells with rapamycin and co-cultured with macrophage-like cells. Our results showed that TRAF2 depletion led to decreased CD206 and CD301 mRNA, the effect ameliorated at least partially by rapamycin treatment in these cells (Fig. 7E). To further strengthen our findings that TRAF2-dependent autophagy regulates macrophage polarization, we treated TRAF2-depleted ccRCC cells with rapamycin and subsequently co-cultured with HUVECs. Our tube formation assays showed that autophagy activator reversed the effect of TRAF2 depletion on the inhibition of M2 macrophage polarization (Fig. 7F-G). Our Western Blot assays showed that autophagy activator partially reversed the decrease in VEGFA protein caused by TRAF2 depletion (Fig. 7H). Similarly, our migration assays showed that autophagy activator partially reversed the weakened macrophage migration induced by TRAF2 depletion (Fig. 7I). Therefore, our results suggest that TRAF2-induced polarization of M2 macrophages is dependent on autophagy.

### TRAF2 is critical for ccRCC tumorigenesis and metastasis in vivo

**Fig. 8:**
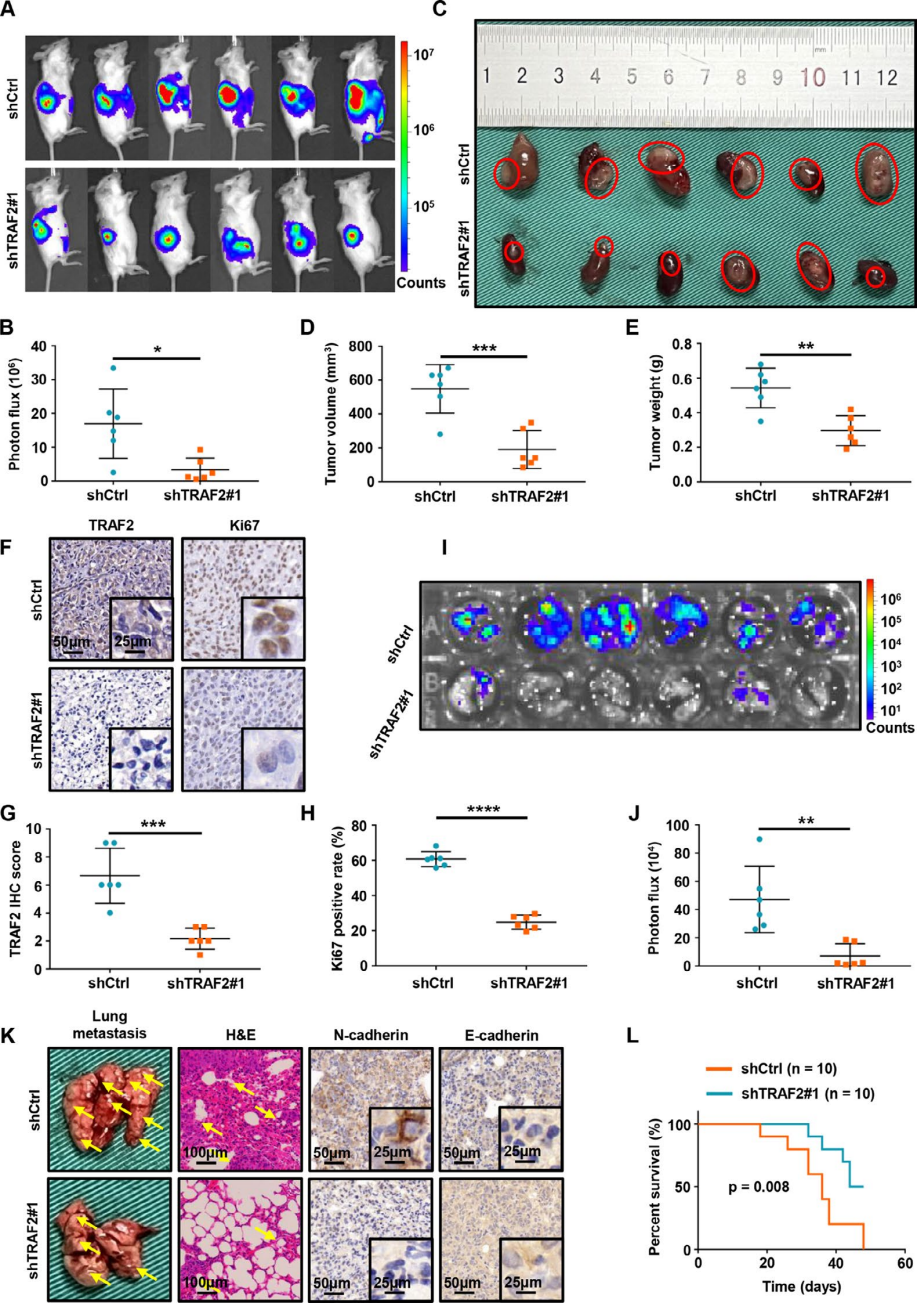
TRAF2 is critical for ccRCC tumorigenesis and metastasis in vivo. (**A**) Images of the orthotopic transplantation mouse model (shCtrl and shTRAF2#1). (**B**) Quantification of the orthotopic tumor photon flux. (**C**) Image of orthotopic transplanted tumors from shCtrl and shTRAF2#1 groups. (**D**, **E**) Tumors were removed 8 weeks after orthotopic transplantation, followed by volume calculation and weight measurement. (**F**) Representative IHC staining images of TRAF2 and Ki67. (**G**, **H**) Quantification of TRAF2 expression and Ki67 positive rate. (**I**, **J**) Representative images of the orthotopic transplantation lung metastasis mouse model model (shCtrl and shTRAF2#1) and the analysis of the lung metastasis photon flux. (**K**) Representative images of mouse lung gross specimens, hematoxylin and eosin (H&E) staining, and IHC staining for E/N-cadherin. (**L**) The survival of mice orthotopically transplanted with OSRC2-luciferase cells expressing shCtrl or shTRAF2#1 was documented (n = 10 mice per group, p = 0.008). Error bars, SEM; *, p < 0.05; **, p < 0.01; ***, p < 0.001, ****, p < 0.0001

Motivated by our in intro phenotype of TRAF2 depletion, we next aimed to determine the effect of TRAF2 on ccRCC tumor growth and metastasis in vivo. First, we infected the previously constructed TRAF2-depleted OSRC2 cells with firefly luciferase vectors and injected these cells orthotopically into kidney capsules of B-NDG mice. After kidney tumor growth was established, we monitored the tumor growth rate of ccRCC cells using a small animal bioluminescence imaging system. Our small animal bioluminescent imaging monitoring results showed that TRAF2 depletion led to decreased renal tumor growth (Fig. 8A-B). When mice were necropsied, mice in the TRAF2-depleted group showed reduced tumor burden compared with control mice (Fig. 8C). We found that TRAF2 depletion decreased tumor growth (Fig. 8D), tumor weight (Fig. 8E), and tumor cell Ki67 positive rate (Fig. 8F-H) in mice models of orthotopic transplantation. To determine whether TRAF2 depletion affects pulmonary metastasis of ccRCC in vivo, we measured the intensity of bioluminescence in lung and found that depletion of TRAF2 inhibited lung metastasis (Fig. 8I-J). We further detected the expression of TRAF2 in lung metastases of shCtl and shTRAF2#1 mice, and the results showed that TRAF2 decreased in lung metastases of shTRAF2#1 mice (Fig. S4C). We detected the expression of TRAF2, CD31, LC3B and CD206 in mouse samples by IHC. Our results suggested that the expression of TRAF2, CD31, and CD206 were decreased, while the expression of LC3 increased in the shTRAF2#1 group (Fig. S4D-E). Similarly, our H&E staining results showed that TRAF2 depletion decreased lung metastases and N-cadherin but increased the E-cadherin compared with the control group (Fig. 8K, Fig. S4F). Importantly, TRAF2 depletion in ccRCC cells prolonged the OS in mice with lung metastasis (p = 0.008, Fig. 8L).

To evaluate the clinical significance of TRAF2/autophagy signaling axis in promoting M2-polarized tumor-associated macrophage infiltration, angiogenesis, and cancer progression, we detected the expression of TRAF2, CD31, LC3, and CD206 in cancerous tissues of patients with ccRCC. We classified samples into TRAF2-low and TRAF2-high groups and determined their relevance in ccRCC. We found the expression of TRAF2 was positively correlated with CD31 and CD206, while TRAF2 was negatively correlated with LC3 (Fig. S4G-J). Together, our results reveal that TRAF2 plays a key role as a promotor of ccRCC growth and metastasis.

## Discussion

In our study, we identified a cluster of genes related to M2 macrophages through biological information analysis. Among them, those associated with ccRCC progression were used to construct an effective patient risk layered model. Based on this risk-stratified model and the clinicopathological data of patients, we constructed a nomograph for predicting patient survival, which is expected to instruct more reasonable diagnosis and treatment decisions and comprehensive management of ccRCC patients. We found that TRAF2, one of the genes that constitute the stratified model, is a potential therapeutic target for ccRCC. We found that TRAF2 depletion suppressed ccRCC growth and progression, and this function was dependent on autophagy inactivation-induced polarization of M2 macrophages. Thus, we revealed a novel TRAF2-autophagy-M2 macrophage oncogenic signaling axis in ccRCC.

In recent years, the key role of TAMs in human tumors has received increasing attention. As the main component of TME immune cells, TAMs promote tumorigenesis [26], metastasis [27], tumor angiogenesis [28], immunosuppression [29] and drug resistance [30] by secreting signaling molecules and cooperating with inflammatory factors. TAMs are stimulated by specific TME signals and shows remarkable plasticity, which is an important entry point to investigate tumor therapeutic targets. In pancreatic and breast cancer, the polarization and recruitment of M2 macrophages is regulated by the POU1F1/CXCL12/CXCR4 signaling axis to promote tumor metastasis [31, 32]. In colorectal cancer, TCF4 promotes liver metastasis by recruiting TAMs and polarization of M2 macrophages via the CCL2-CCR2 axis [33]. In esophageal squamous cell carcinoma (ESCC), IL-32 internalized by macrophages leads to the polarization of M2 macrophages through the FAK-STAT3 pathway, thereby promoting lung metastasis of ESCC [34]. Together with these studies, our findings undoubtedly increase the understanding of the mechanisms by which TAMs regulate tumors and provide new ideas for the development of potential therapeutic targets.

TRAF2-mediated regulation of TME involves multiple signaling pathways. TRAF2 forms a complex with TRAF3 that increased TMAs recruitment mediated by USP17, thereby enhancing stemness and inflammation in lung cancer cells [13]. Recruitment of TRAF2 by IREα activates the NF-κB signaling pathway, leading M2 macrophages to redirect to marker genes encoding M1 macrophages, thereby increasing the therapeutic response of PD-1 inhibitors [35]. Jin et al. revealed that TRAF2 can mediate the proteasome degradation of proinflammatory transcription factors IRF5 and c-Rel, leading to increased macrophage polarization and tumor growth [36]. In acute myeloid leukemia, the TRAF2–cFLIP–NF-κB signaling axis regulates T-cell antitumor immune responses and maintains AML progression [37].In the present study, we found that TRAF2 silencing reduced macrophage polarization towards M2-type, and these findings are consistent with the tumorigenic phenotype of TRAF2 in ccRCC. Moreover, our findings undoubtedly deepen the understanding of how TRAF2 regulates tumor progression through the TME.

TRAF2 is involved in many cellular processes including activation of the NF-κB pathway [37, 38], cell death [39], and autophagy [40]. One of the main biological functions of TRAF2 if to transmit activation signals from cell surface receptors to the IκB kinase complex, which is a key event in the activation process of the NF-κB canonical signaling pathway [38]. TRAF2 recruits and activates the E3 ligases cIAP1 and cIAP2, and cIAPs in turn ubiquitinate TRAF2 at K63, promoting the formation of anchor points for the linear ubiquitin assembly complex. Ubiquitin chains catalyzed by the linear ubiquitin assembly complex provide key binding sites for the basic modulator of the NF-κB signaling pathway [41]. TRAF2-mediated NF-κB activity undoubtedly provides new ideas for identifying new therapeutic targets. For example, the interaction between TRAF2 and LIRB3 is depleted by overactivated NF-κB activation, and the signaling axis is antagonized to inhibit the progression of AML, making targeting LILRB3 a potential intervention option [37]. Xu et al. found that TRAF2 plays an irreplaceable role in the regulation of cell homeostasis after inhibiting cell death. The ubiquitination of beclin 1 mediated by TRAF2 and cIAPs was inhibited by TRADD, resulting in decreased autophagic flux. Small molecule ICCB-19 or Apt-1 can restore homeostasis and delay cell apoptosis by inhibiting TRADD and activating autophagy [40]. The findings of our study reveal that TRAF2 promotes tumor growth and progression through autophagy activation-mediated macrophage M2 polarization, which undoubtedly expands our understanding of TRAF2 regulatory pathways.

Apostatin-1 is a novel TRADD inhibitor that binds to the pocket on the N-terminal TRAF2-binding domain of TRADD. Previous studies reported that Apostatin-1 could inhibit bortezomib-induced apoptosis and RIPK1-dependent apoptosis [40]. Additionally, Birinapant is a SMAC simulator that potently antagonizes XIAP and cIAP1. Birinapant was found to target TRAF2-related cIAP, activate caspase-8 and induce tumor cell death by eliminating TNF-induced NF-κB activation [42]. However, none of these inhibitors directly targets TRAF2. Therefore, due to the lack of TRAF2-specific inhibitors at this stage, we were unable to investigate the inhibitory effect of targeting TRAF2 on tumors in vivo studies. Nonetheless, our study may facilitate the development of TRAF2-specific inhibitors, which will hopefully benefit patients with advanced ccRCC.

## Conclusion

In summary, the prognosis prediction model we constructed has the ability to accurately and stably predict the prognosis of patients, which is expected to promote the improvement of efficacy evaluation and comprehensive management of ccRCC patients. Moreover, our findings reveal that the TRAF2/M2 macrophage/autophagy axis plays a key regulatory role in the malignant progression of ccRCC, and suggest that TRAF2 is a potential novel therapeutic target for advanced ccRCC.

## Electronic supplementary material

Below is the link to the electronic supplementary material.

Supplementary Material 1

Supplementary Material 2

## Data Availability

All data generated or analyzed during this study are included in this published article and its supplementary information files.

## Abbreviations

ccRCC: Clear cell renal cell carcinoma
TME: Tumor microenvironment
TAMs: Tumor-associated macrophages
IL-6: Interleukin 6
IFN-γ: Interferon-γ
TGF-β: Transforming growth factor-β
TNF: Tumor necrosis factor
TRAF2: Tumor necrosis factor receptor-associated factor-2
NF-κB: Nuclear factor kappa B
MAPK: Microtubule-associated protein kinase
M2 MRGs: M2 Macrophages-related genes
WGCNA: Weighted gene co-expression network analysis
LASSO: Least absolute shrinkage and selection operator
ROC: Receiver operating characteristic
AUC: Area under the curve
OS: Overall survival
ATCC: American Type Cultrue Collection
IHC: Immunohistochemistry
H&E: Hematoxylin and eosin
TEM: Transmission electron microscopy
GO: Gene ontology
DEGs: Differentially expressed genes
CCK-8: Cell Counting Kit-8
FC: Flow cytometry
shRNAs: Short hairpin RNAs
2/3D: Two/three-dimensional
