# Supplementary material for: TRAF2 promotes M2-polarized tumor-associated macrophage infiltration, angiogenesis and cancer progression by inhibiting autophagy in clear cell renal cell carcinoma
Source: J Exp Clin Cancer Res. 2023 Jul 6;42:159. doi: 10.1186/s13046-023-02742-w (PMC10324183; doi:10.1186/s13046-023-02742-w)
Supplement: Supplementary file 2 — Supplementary Material 2 [file 13046_2023_2742_MOESM2_ESM.docx]

**Supplementary Material**

**TRAF2 promotes M2-polarized tumor-associated macrophage infiltration, angiogenesis and cancer progression by inhibiting autophagy in clear cell renal cell carcinoma**

**Table S1.** The proportion of tumor-infiltrating immune cell subtypes in each sample.

**Table S2.** M2 macrophage-related genes in ccRCC.

**Table S3.** The clinicopathologic characteristics of 16 ccRCC patients.

**Figure S1.** The regulatory network between M2 MRGs and M2 macrophages.

**Figure S2.** TRAF2 promotes cell proliferation, migration, and invasion in ccRCC cells.

**Figure S3.** TRAF2 promotes ccRCC progression via regulating macrophage polarization, migration, and angiogenesis.

**Figure S4.** TRAF2 is critical for ccRCC tumorigenesis and metastasis.

**Table S1** and **Table S2** are presented in separate attachments.

**Table S3** The clinicopathologic characteristics of 16 ccRCC patients

| **Variables** | n (%) |
| --- | --- |
| Gender |  |
| Male | 8 (50.0) |
| Female | 8 (50.0) |
| Age (years) |  |
| ≤ 60 | 10 (62.5) |
| > 60 | 6 (37.5) |
| Stage _T |  |
| T1+T2 | 9 (56.3) |
| T3+T4 | 7 (43.7) |
| Stage _N |  |
| N0 | 14 (87.5) |
| N1 | 2 (12.5) |
| Stage _M |  |
| M0 | 12 (75.0) |
| M1 | 4 (25.0) |
| TNM stage |  |
| Ⅰ+Ⅱ | 5 (31.3) |
| Ⅲ+Ⅳ | 11 (68.7) |
| Fuhrman grade |  |
| 1+2 | 8 (50.0) |
| 3+4 | 8 (50.0) |
| Nephrectomy |  |
| Nephron-sparing surgery | 6 (37.5) |
| Radical nephrectomy | 10 (62.5) |


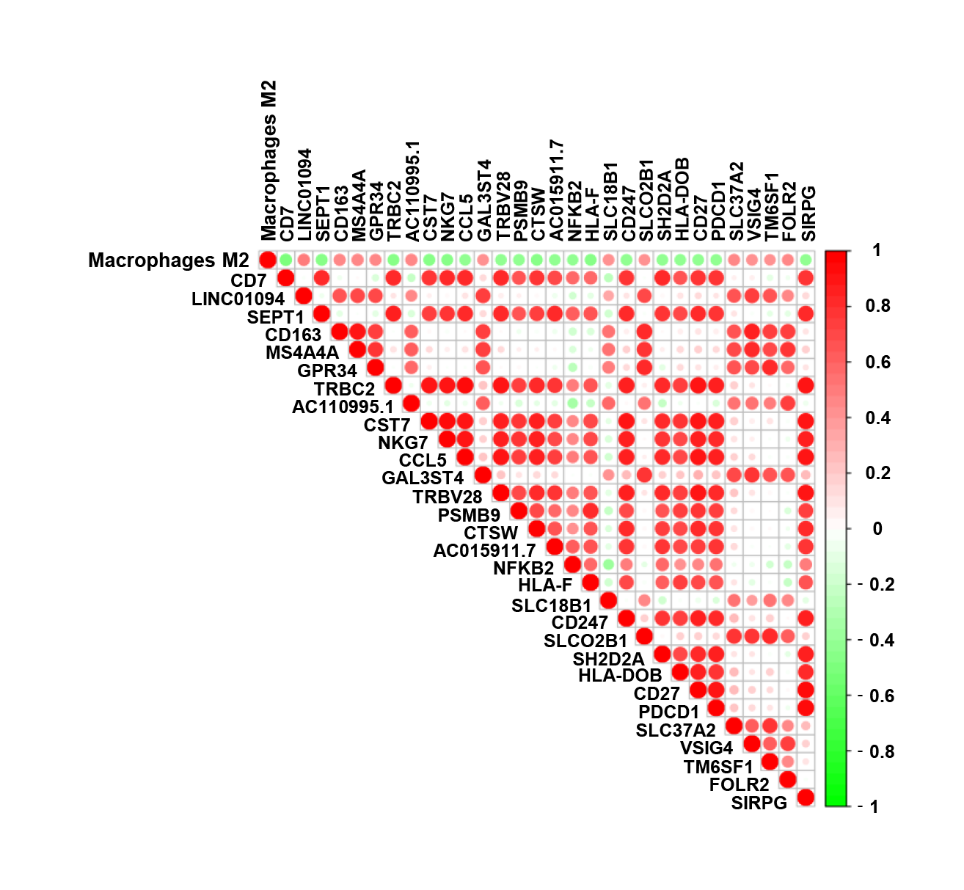


**Figure S1.** The regulatory network between M2 MRGs and M2 macrophages.


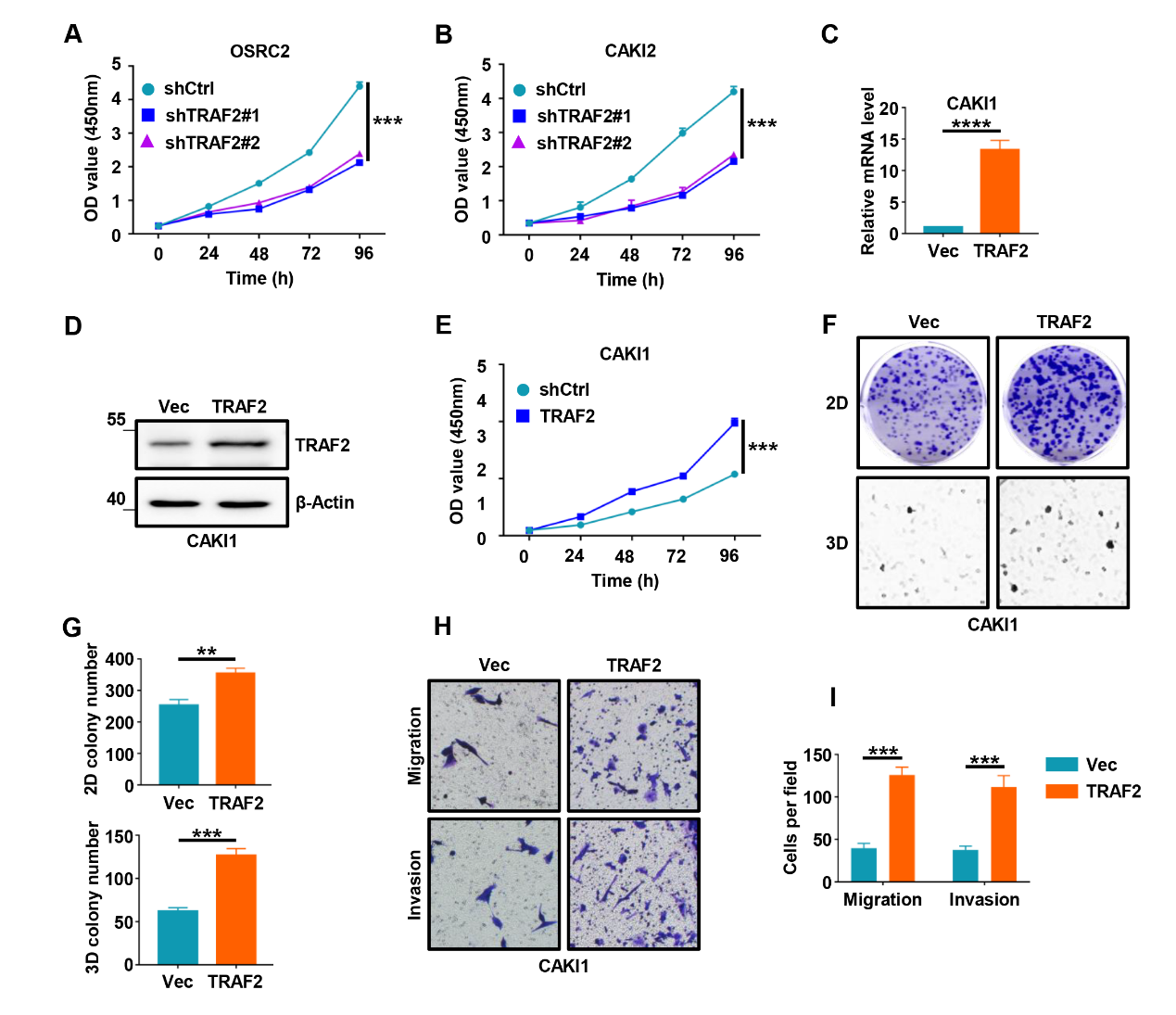


**Figure S2.** TRAF2 promotes cell proliferation, migration, and invasion in ccRCC cells. (A-B) Viability of OSRC2 and CAKI2 cells (shCtrl, shTRAF2#1, and shTRAF2#2) detected by the CCK-8 assay. (C-D) Stable overexpression of TRAF2 effectively increased its mRNA and protein levels in CAKI1 cells. (E) Viability of CAKI1 cells (vector and TRAF2) detected by the CCK-8 assay. (F-G) Representative colony formation assay and 3D soft agar growth (F) and quantification of colony number (G). (H-I) Representative cell migration and invasion pictures (H) and quantification of cell migration and invasion (I). Error bars, SEM; **, p < 0.01; ***, p < 0.001, ****, p < 0.0001.


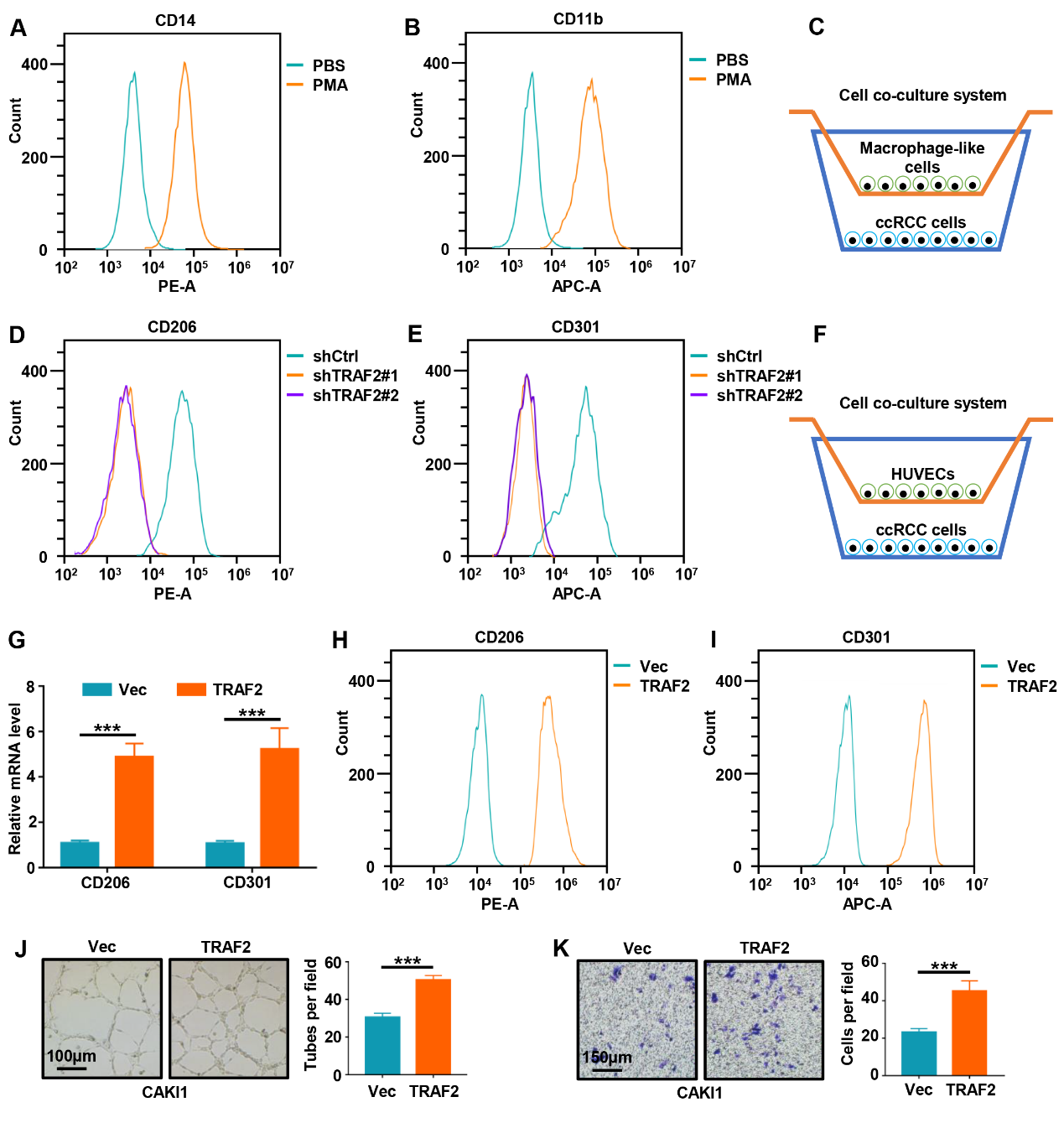


**Figure S3.** TRAF2 promotes ccRCC progression via regulating macrophage polarization, migration, and angiogenesis. (A-B) Cell surface CD14 and CD11b level were detected by flow cytometry in THP-1 cells treated with PMA (200 nM, 48 hours). (C) Co-culture system of macrophage-like cells and OSRC2 cells (shCtrl, shTRAF2#1, and shTRAF2#2). (D-E) Cell surface CD206 and CD301 level were detected by flow cytometry in macrophage-like cells co-cultured with shCtrl or TRAF2 depleted OSRC2 cells. (F) Co-culture system of HUVECs and OSRC2 and CAKI2 cells (shCtrl, shTRAF2#1, and shTRAF2#2). (G) RT-qPCR analysis was used to detect the markers of M2 macrophages (CD206 and CD301) mRNA expression changes in macrophage-like cells co-cultured with vector or TRAF2 overexpressed OSRC2 cells. (H-I) Cell surface CD206 and CD301 level were detected by flow cytometry in macrophage-like cells co-cultured with vector or TRAF2 overexpressed OSRC2 cells. (J) Representative tube-formation pictures of in vitro angiogenesis (left panel) and quantification of the node branches (right panel). (K) Representative cell migration pictures of macrophages (left panel) and quantification of cell migration (right panel). Error bars, SEM; ***, p < 0.001.


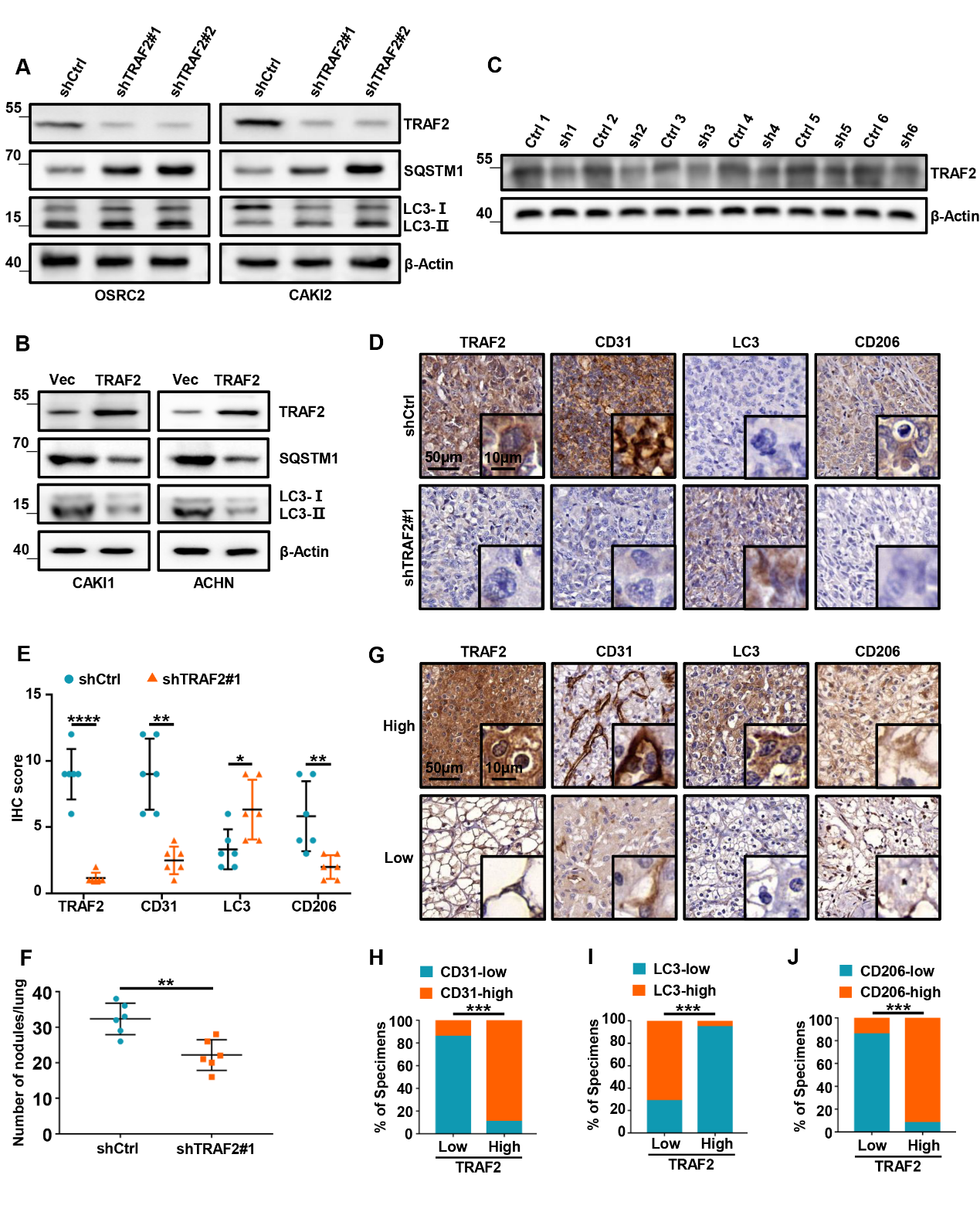


**Figure S4.** TRAF2 is critical for ccRCC tumorigenesis and metastasis. (A-B) Western Blot analysis of the effect of TRAF2 depletion and overexpression on autophagic flux. (C) Western Blot assay was used to detect the expression pattern of TRAF2 in lung metastases (“Ctrl”=shCtrl, “sh”=shTRAF2#1) (n=6). (D-E) Representative IHC staining images of TRAF2, CD31, LC3, and CD206 in shCtrl and shTRAF2#1 mice (D), and quantitative analysis between TRAF2 level and CD31, LC3, and CD206. (F) Quantification of lung metastases. (G) Representative IHC staining images of high and low levels of TRAF2, CD31, LC3, and CD206 in ccRCC patient samples. (H-J) TRAF2 levels were significantly correlated with CD31, LC3, and CD206 in ccRCC patient samples (n=45).
